# Making Sense of the Epigenome Using Data Integration Approaches

**DOI:** 10.3389/fphar.2019.00126

**Published:** 2019-02-19

**Authors:** Emma Cazaly, Joseph Saad, Wenyu Wang, Caroline Heckman, Miina Ollikainen, Jing Tang

**Affiliations:** ^1^Institute for Molecular Medicine Finland, Helsinki Institute of Life Science, University of Helsinki, Helsinki, Finland; ^2^Department of Public Health, University of Helsinki, Helsinki, Finland; ^3^Department of Mathematics and Statistics, University of Turku, Turku, Finland; ^4^Research Program in Systems Oncology, Faculty of Medicine, University of Helsinki, Helsinki, Finland

**Keywords:** epigenetics, data integration, functional annotation, drug discovery, data resources, profiling techniques

## Abstract

Epigenetic research involves examining the mitotically heritable processes that regulate gene expression, independent of changes in the DNA sequence. Recent technical advances such as whole-genome bisulfite sequencing and affordable epigenomic array-based technologies, allow researchers to measure epigenetic profiles of large cohorts at a genome-wide level, generating comprehensive high-dimensional datasets that may contain important information for disease development and treatment opportunities. The epigenomic profile for a certain disease is often a result of the complex interplay between multiple genetic and environmental factors, which poses an enormous challenge to visualize and interpret these data. Furthermore, due to the dynamic nature of the epigenome, it is critical to determine causal relationships from the many correlated associations. In this review we provide an overview of recent data analysis approaches to integrate various omics layers to understand epigenetic mechanisms of complex diseases, such as obesity and cancer. We discuss the following topics: (i) advantages and limitations of major epigenetic profiling techniques, (ii) resources for standardization, annotation and harmonization of epigenetic data, and (iii) statistical methods and machine learning methods for establishing data-driven hypotheses of key regulatory mechanisms. Finally, we discuss the future directions for data integration that shall facilitate the discovery of epigenetic-based biomarkers and therapies.

## Introduction

Complex diseases and traits have a genetic background, yet the final phenotypic outcome largely depends on an individual’s environment and lifestyle, and genomic studies have thus far explained only a small fraction of the inherited risk of many complex diseases (Eichler et al., 2010). This missing heritability may in part be explained by the contribution of epigenetic variation to complex diseases. Moreover, the majority of genetic variants associated with a disease risk are located at non-coding regions of the genome, suggesting that these SNPs point to genomic regions with a downstream regulatory role. It is well-established that cells regulate gene expression during multiple stages of transcription and translation, predominantly through chromatin packaging (Holliday, 2006). Chromatin is a complex of DNA and DNA binding proteins that control the packaging of DNA and thereby affect the access of transcription factors to the regulatory regions of genes. This process is regulated by two epigenetic mechanisms: dynamic DNA methylation and post-translational modifications of DNA binding histone proteins.

DNA methylation plays an important role in silencing tissue-specific genes, imprinted genes and repetitive elements (Walsh et al., 1998; Fouse et al., 2008). DNA methylation in human cells occurs predominantly at the cytosine of a cytosine-guanine pair (CpG dinucleotide), where a methyl group is covalently attached to the carbon 5 position. In the human genome there are approximately 28 million CpG dinucleotides, accounting for 1% of the whole genome. Of these, 60 to 90% are methylated, while the majority of unmethylated sites cluster non-randomly in regions called CpG islands (CGIs). CGIs co-localize to the promoter region of up to 70% of human genes (Illingworth and Bird, 2009). In general, unmethylated CGIs are associated with transcriptionally permissive chromatin and gene expression. During normal development and in certain disease states, particularly in cancer, these CGIs can become methylated, leading to inhibition of transcription factor binding and gene repression.

In addition to DNA methylation (5mC), DNA hydroxymethylation (5hmC) is another essential epigenetic modification in cells. Hydroxymethylation is the primary product of the oxidation of 5-methylcytosine by the ten-eleven translocation (TET) enzymes. In this process methylated cytosine (5mC) is first oxidized into 5-hydroxymethylcytosine (5hmC), then to 5-formylcytosine and to 5-carboxylcytosine (5caC). These are removed by thymine DNA glycosylase and replaced by unmethylated cytosine by base excision repair. However, hydroxymethylation is not merely an intermediate of the dynamic demethylation process but actually a temporarily stable epigenetic modification of DNA (Globisch et al., 2010). Hydroxymethylated cytosines are enriched at the promoters and enhancers of developmental genes, and they correlate positively with gene expression during cell lineage commitment in early development. In addition, hydroxymethylation is present in gene bodies of actively transcribed genes (Colquitt et al., 2013; Tsagaratou et al., 2014; Nestor et al., 2016). Hydroxymethylation is less abundant than DNA methylation, and its abundance varies between tissues and cell types. It is more abundant in embryonic stem cells (Ito et al., 2010), and human brain tissue (0.67%), kidney (0.38%), colon (0.45%), rectum (0.57%), and liver (0.46%), while low or very low in human lung, breast and placenta (Li and Liu, 2011). The abundance of hydroxymethylation seems to be inversely correlated with the proliferation rate of a cell (Kriaucionis and Heintz, 2009; Bachman et al., 2014). The dynamic interplay between DNA methylation and hydroxymethylation is presumably important for maintaining normal gene expression patterns in a cell, however, the causes and consequences of the imbalance between these two DNA modifications is still to be understood.

In contrast to DNA methylation and hydroxymethylation, which are set *de novo* at early embryogenesis and maintained during DNA replication, histone modifications are post-translational changes. They act to remodel the chromatin structure and regulate gene expression through chromatin accessibility (ENCODE Project Consortium, 2012). Histone modifications are the largest category of chromatin modifications identified so far, with 15 known chemical modifications at more than 130 sites on 5 canonical histones and on around 30 histone variants. Specific histone modification patterns often correlate with known functional genomic elements. For example, H3K9me3 and H3K27me3 are associated with inactive promoters; while H3K4me3 and H3K27ac are shown to be enriched in active enhancers and promoters (Karlic et al., 2010; Zhou V.W. et al., 2011). However, the theoretical number of all possible histone – modification combinations is huge, particularly when compared to the extremely limited knowledge on their functional roles.

An additional layer of epigenetic regulation is derived from non-coding RNA (ncRNA), which is transcribed from DNA but not translated into protein. NcRNA ranges from very small 22 nucleotide microRNA molecules (miRNA), to transcripts longer than 200 nucleotides (lncRNA). NcRNAs play a role in translation, splicing, DNA replication and gene regulation, particularly through miRNA directed downregulation of gene expression (Valencia-Sanchez et al., 2006). NcRNAs are most widely studied in the context of cancer, where they have been identified in the tumor suppressor or oncogenic processes of all major cancers (Anastasiadou et al., 2018). The techniques for measuring ncRNA are similar to other transcriptomic techniques, predominantly involving deep sequencing approaches (Veneziano et al., 2016). In recent years it has become apparent that there is a coordinated interaction between ncRNA and other epigenetic marks, the extent of which is yet to be fully realized (Ferreira and Esteller, 2018). The discovery of more than 100 post-transcriptional modifications to ncRNA, such as methylated adenines and cytosines, is providing further insight into the interaction between these different epigenetic layers (Romano et al., 2018). For the latest advances in the ncRNA biology, we refer the reader to the special series in Nature Reviews Genetics, January 1st 2018^1^.

DNA methylation (referring to both 5mC and 5hmC from here on), histone modifications and ncRNA respond to genetic and environmental effects and thereby alter gene expression, providing biological mechanisms for the development of common diseases. Therefore, epigenetic mechanisms are key to understanding disease progression and discovering new treatment targets (Lord and Cruchaga, 2014). As one of the more recent omics fields, epigenomics has experienced rapid growth in the past decade, providing novel insights to many areas of cell biology. Recent developments in microarray technology have made the generation of genome-wide epigenetic data feasible in large populations (Pidsley et al., 2016). As such, epigenome-wide association studies (EWASs) have become an important component of omics-driven approaches to investigate the origin of common human traits and diseases (Lappalainen and Greally, 2017).

**FIGURE 1:**
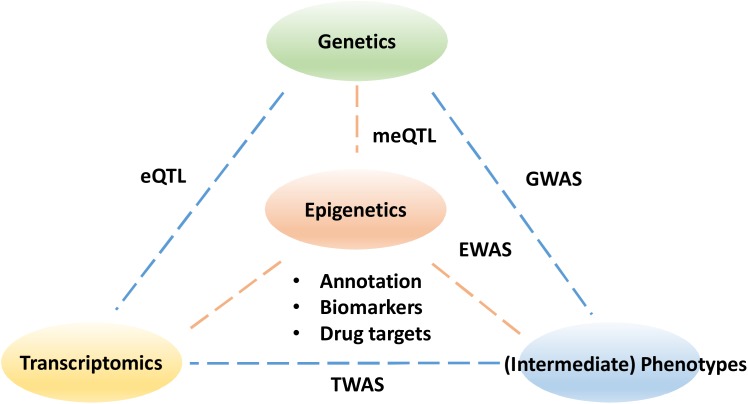
The pillars to understanding the functional impact of epigenetics data. The epigenetic links need to be made with sequence variants in genetics as well as changes in transcriptomics. Understanding the impact of epigenetics on intermediate phenotypes for example metabolomics and proteomics may ultimately help explain the disease etiology and help drug discovery. GWASs, genome-wide association studies; EWASs, epigenome wide association studies; meQTL, methylation quantitative trait loci; eQTL, expression quantitative trait loci; TWAS, transcriptome-wide association study.

Despite the tremendous potential to improve our understanding of disease progression and treatment, epigenetics has yet to become fully utilized in clinical applications. Similar to transcriptomics, epigenetic profiles are continuous, dynamic and tissue-specific. As ever more epigenetic data are generated with advances in high-throughput sequencing and microarray technologies, the challenges now become developing data analysis approaches to facilitate the identification of coordinated epigenetic changes and interpretation of their functional consequences in normal development and disease. For example, an effective data annotation protocol is needed for a community-driven data standardization to improve the replicability of epigenetic findings (Carter et al., 2017). In particular, the variation in epigenetics profiles at different time points is yet to be established as a control for the reference in normal populations. Partly due to the lack of appropriate and efficient computational methods, the majority of existing studies focus on a single epigenetic mark in isolation, although the interactions of multiple marks and genotypes exist *in vivo* (Davila-Velderrain et al., 2015). To realize the full potential offered by epigenetics, an interdisciplinary research community is needed to foster effective and robust data integration strategies for combining epigenetics data with other omics data (Figure 1).

In the following sections we will review the recent advances in computational methods and applications for epigenomic analysis and discovery, ranging from databases and software tools for statistical analysis to data integration techniques for functional annotation. We will start by comparing the common epigenomic profiling technologies, before moving on to data annotation and standardization models. We then provide an overview of various data sources leveraged in epigenetic studies and their applications. We describe statistical and machine learning methods to pinpoint epigenetic modifications driving disease, and provide a list of software tools capable of implementing these methods, as well as databases containing epigenomic and other omics data. This catalog provides a comprehensive and practical resource to build data-driven hypotheses for analyzing the functional consequences of epigenetic marks. Finally, we provide representative examples of profiling epigenetics in disease states and its significance in biomarker and drug discovery.

## Epigenetic Profiling Techniques

Epigenetic analysis techniques can be broadly classified as *typing*, involving a small number of loci across many samples, or *profiling* that can be extended to epigenome-wide analysis. The end-point measurements from these methods often reflect a proportion or ratio of chromatin with epigenetic marks compared to the total chromatin. Within these categories, various sequencing, microarray and antibody based methodologies are employed to examine the different aspects of epigenetic regulation, including DNA methylation, chromatin accessibility and histone modifications. Epigenetic data generated from these techniques require different pre-processing steps depending on the methodology employed. For example, array-based DNA methylation analysis requires extensive within and between array normalization, preprocessing and integration across platforms (Fortin et al., 2017), while bisulfite sequencing can be processed with a relatively standardized sequence trimming and alignment pipeline (Wreczycka et al., 2017). Further complications include the feasibility of using epigenetic profiles derived from blood as a proxy for other less accessible tissue types (Houseman et al., 2015), and controlling for tumor purity in cancer studies (Zheng et al., 2017). Here, we summarize the most common epigenetic profiling techniques and compare their advantages and limitations (Tables 1, 2).

**Table 1:** Summary of major profiling techniques for DNA methylation.

Technique	Method	Advantages	Limitations
Whole-Genome Bisulfite Sequencing (WGBS)	Bisulfite converted DNA is amplified and sequenced	Genome-wide, single nucleotide resolution	Costly and computationally intensive
Reduced-Representation Bisulfite Sequencing (RRBS)	Methylation-insensitive restriction enzymes digest DNA, enriching for CpG regions	Cheaper than WGBS with relatively high coverage	Enzymatic digestion covers most but not all CpG sites
Pyrosequencing	DNA is bisulfite converted, amplified, with the ratio of C/T nucleotides measured	Genome-wide or targeted, single nucleotide resolution. Allele-specific primers	Relatively expensive
Methylated DNA Immunoprecipitation (MeDip)	Methylated DNA is enriched by immunoprecipitation followed by sequencing or microarray analysis	Random fragmentation by sonication avoids restriction enzyme bias	Varying CpG density can confound methylation estimates
Methylation Sensitive Restriction Enzyme Sequencing (MSRE/MRE-Seq or Methyl-seq)	Unmethylated DNA is restriction enzyme digested while methylated DNA is amplified	No bisulfite conversion bias	DNA may be partially digested, limited coverage
Combined Bisulfite Restriction Analysis (COBRA)	Bisulfite converted DNA is amplified and restriction enzyme digested	Simple, fast, inexpensive, works on FFPE-treated DNA	DNA may be partially digested, limited coverage
Methylation Specific PCR	Bisulfite converted DNA is amplified with methylation specific primers	Simple and inexpensive	Purely qualitative
High Resolution Melt Analysis (HRM)	Bisulfite converted DNA is amplified by q-PCR	Most sensitive method for determining methylation at a specific region	Single base resolution not possible
Illumina MethylationEPIC BeadChip Microarray (previously 450k, 27k)	Bisulfite (or oxidized + bisulfite) converted DNA is interrogated on a microarray chip	Relatively simple and inexpensive. Extremely popular	Data has limited coverage and requires pre-processing
Global DNA Methylation	Methods include LINE1, Alu, LUMA, HPLC-UV	Relatively inexpensive	Does not identify differentially methylated regions
Tet-assisted Bisulfite Sequencing (TAB-seq)	5hmC is protected then oxidized to 5caC then uracil by TET	Differentiation between 5mC and 5hmC at single base resolution	Sensitivity and specificity depends on sequencing depth
Oxidative Bisulfite Sequencing (OxBis)	DNA is oxidized then bisulfite converted to 5fC and subsequently uracil	Quantitative genome-wide coverage	Bias to regions of low 5mC. Must be performed in parallel with bisulfite techniques
APOBEC-coupled epigenetic sequencing (ACE-seq)	Non-destructive DNA deaminase enzymes discriminate between 5hmC and 5mC	Genome-wide, single nucleotide resolution. Very low DNA input required	Not yet extensively tested
Hydroxymethylated DNA Immunoprecipitation (hMeDIP)	Immunoprecipitation and sequencing of hydroxymethylated DNA	Simple and inexpensive	Only semi quantitative and bias to regions of low 5hmC

**Table 2:** Summary of major profiling techniques for Chromatin Accessibility and Histone modifications.

Technique	Method	Advantages	Limitations
Chromatin Immunoprecipitation (ChIP)	Couples highly specific antibodies for DNA-binding proteins with sequencing, microarrays or PCR	Detect DNA associated proteins and histone modifications	Requires intact cells and chromatin
Digital DNase	Enzymes digest nuclease-accessible regions, indicating open chromatin	Maps both nucleosomes and non-histone proteins	High sequencing depth required. Potential actin contamination.
NOMe-seq	Single-molecule, high-resolution nucleosome positioning assay	Maps both DNA methylation and nucleosomes at high resolution	Relies on presence of CpG residues
Assay for Transposase-Accessible Chromatin using sequencing (ATAC-seq)	Measures chromatin accessibility based on Tn5 transposase activity. Maps nucleosomes and non-histone proteins	Simple, fast, low input of cells with single nucleotide resolution	Distance between binding sites may bias results
Chromosome Conformation Capture (3C, 4C, 5C, Hi-C)	Assess spatial organization of chromatin in a cell	Various modified versions	Often lack genome-wide, single nucleotide resolution

## Data Resources for Standardization, Annotation, and Harmonization

Unlike the human genome, the epigenome varies across different cell types and over time. Due to recent efforts in big data consortia, such as the Encyclopedia of DNA Elements (ENCODE) (Davis et al., 2018) and the International Human Epigenome Consortium (IHEC) (Bujold et al., 2016), genome-wide epigenetic reference datasets are now publically available for different cell lineages, tissues, and diseases. Within IHEC, standardization of sample preparation and assay protocols have been benchmarked and implemented across multiple centers, that have been collected from seven international consortia including ENCODE, NIH Roadmap (Bernstein et al., 2010), Blueprint (Martens and Stunnenberg, 2013) and others across Europe, North America, and Asia. Furthermore, efficient data portal infrastructure has provided powerful tools for interactive exploration and annotation of the resulting datasets at a genome-wide scale, encompassing over 800 reference epigenomes for different tissues and conditions. Such a community-driven profiling effort has provided rich resources and tools for future epigenetic data mining and functional annotation. More recently, these datasets have been made available via the Human Epigenome Browser (Zhou X. et al., 2011), providing the visualization tools similar to the UCSC Genome Browser (Kent et al., 2002). Here, we list the common data repositories and their visualization tools (Table 3).

**Table 3:** Epigenetic data repositories and browsers.

Consortia and resources	Data availability	URLs
The International Human Epigenome Consortium (IHEC)	Reference epigenomes generated by NIH Roadmap, ENCODE, CEEHRC, BLUEPRINT, DEEP, AMED/CREST, and KEP	IHEC Data Portal http://epigenomesportal.ca/ihec
NIH Roadmap Epigenomics	Maps of histone modifications, chromatin accessibility, DNA methylation and mRNA Expression in stem cells and primary *ex vivo* human tissues	VizHub http://vizhub.wustl.edu
Canadian Epigenetics, Environment and Health Research Consortium (CEEHRC) Network	Reference epigenomes including histone modifications, DNA methylation, mRNA and miRNA of human cancer and normal cells	CEEHRC Data http://www.epigenomes.ca/site-data Software Tools http://www.epigenomes.ca/site-data Software Tools http://www.epigenomes.ca/tools-and-software
BLUEPRINT Epigenome	Reference epigenomes of human normal and malignant hematopoietic cells	BLUEPRINT Portal http://blueprint-data.bsc.es
The German epigenome programme (DEEP)	Reference epigenomes of human cells and tissues in normal and complex disease states	DEEP Data Portal http://deep.dkfz.de
IHEC Team Japan (AMED-CREST)	Reference epigenomes of human gastrointestinal epithelial cells, vascular endothelial cells and cells of reproductive organs	IHEC Data Portal http://epigenomesportal.ca/ihec
Korea Epigenome Project (KEP)	Reference epigenome map for common complex diseases	IHEC Data Portal http://epigenomesportal.ca/ihec
DeepBlue	Epigenomic data server for storing and working with genomic and epigenomic data. Collection of over 30,000 experiment files from the main epigenome mapping projects available. Uploading own data allowed	DeepBlue server http://deepblue.mpi-inf.mpg.de
Allelic Epigenome Project	Allelic DNA methylome, histone modifications, and transcriptome in human cells and tissues	Genboree http://genboree.org/genboreeKB/projects/allelic-epigenome
GTEx	Genotype and expression profiles in different tissues enabling eQTL studies	GTEx Portal http://www.gtexportal.org
BRAINEAC	Brain eQTL Almanac provides genotype and expression profile across 10 brain regions	BRAINEAC http://braineac.org
MQTLdb	Methylation and genotype data on mother-child pairs providing access to meQTL mapping across five different stages of life	mQTL Database http://www.mqtldb.org
Fetal brain meQTLs	Epigenome-wide significant meQTLs observed in fetal brain	Fetal Brain meQTL http://epigenetics.essex.ac.uk/mQTL
Pancan-meQTL	Database of *cis-* and *trans-* meQTLs across 23 cancer types from The Cancer Genome Atlas	Pancan-meQTL http://bioinfo.life.hust.edu.cn/Pancan-meQTL
Epigenome Browser	UCSC genome browser with tracks from ENCODE project	UCSC Epigenome Browser http://www.epigenomebrowser.org
WashU Epigenome Browser	Web browser with tracks from ENCODE and Roadmap Epigenomics projects	WashU Epigenome Browser http://epigenomegateway.wustl.edu
Ensembl	ENCODE data used in the regulatory build	Ensembl ENCODE https://www.ensembl.org
RMBase	Database listing over 100 RNA modifications	http://rna.sysu.edu.cn/rmbase/

To facilitate the sharing of epigenomic data between different studies, standardization of sample preparation and assay protocols is required. While there are existing recommendations for reporting the minimal information to annotate omics studies such as MIAME for gene expression data and MIAPE for proteomics data, the consensus for the annotation protocol for epigenetics data has yet to be defined. This is partly due to the versatile techniques for various epigenetic features that require distinctive experimental protocols for achieving optimal results (Chervitz et al., 2011). To improve the data interoperability, comparisons of the epigenetics profiling techniques have been initiated by the international consortia. For example, the BLUEPRINT consortium has conducted a systematic comparison of different DNA methylation profiling technologies and reported generally consistent results, whilst also highlighting the higher performance of sequencing-based assays over array-based or antibody-based assays (BLUEPRINT Consortium, 2016). Moreover, informatics approaches such as APIs (Application Programming Interfaces) have been developed to extract data from different repositories in a more efficient manner. One example is the DeepBlue web server, which provides an API for retrieving major epigenetic studies of IHEC (Albrecht et al., 2016). The use of resource description framework (RDF) such as Bio2RDF has also been proposed to allow for the sharing of knowledge to facilitate text mining techniques for information retrieval (Jupp et al., 2014).

## Statistical and Data Integration Methods for Interrogating the Epigenome

As is the case in association studies in other fields, EWAS detect epigenetic marks associated with a certain phenotype. Common epigenetic study designs include case-control studies, cross-sectional or longitudinal cohort studies, and family or twin designs. Logistic regression is commonly used for a case vs. control or binomial phenotype design, while linear regression is employed with continuous phenotypes. Technical and biological covariates are added to the regression models to adjust for confounding factors in the data and methods that control the false discovery rate posed by multiple testing are applied.

The resulting epigenetic profiles can be visualized on appropriate web tools, such as UCSC Genome Browser (Kent et al., 2002), EpiGenome Browser (Zhou X. et al., 2011), or coMET (Martin et al., 2015). While recent advances in epigenetic profiling techniques have made EWAS more cost-efficient and effective, interpreting the results from such epigenomic studies remains a challenge. Without a careful selection of tissues and population samples, many EWAS associations may partly stem from the dynamic and complex nature of the interactions between the different epigenetic layers, or arise from the fact that epigenetic states differ spatially across tissues and cell types as well as during aging. Therefore, there have been significant difficulties inferring the causality of epigenetic marks among a range of genetic, environmental and stochastic factors. A variety of data integration approaches, such as co-mapping and network analysis are currently employed to unravel the complexities of these various epigenetic layers and their interaction with other omics datasets (Hasin et al., 2017).

In this section we discuss data integration approaches for the functional annotation of trait-associated epigenetic hits by the use of knowledge bases, by predicting chromatin states, and by establishing associations with gene expression. Alternatively, the genetic basis of DNA methylation marks can be studied using the meQTL analyses, from which computational tools can be utilized to further identify the potential functional variants. The results of robust associations between genetic variants, epigenetics marks and disease traits can be integrated in the framework of causal modeling, with an aim to dissect causal epigenetic marks from those that are secondary to disease progression. These likely causal epigenetic marks may be further developed into potential disease biomarkers and drug targets upon experimental investigation, for example using epigenome editing techniques.

### Functional Annotation

#### Pathways

Genes and their regulators do not function in isolation, but are organized into pathways and networks. To obtain a more holistic view on the potential functional implications of the EWAS hits, multiple tools on gene ontologies (GOs), pathway and network analysis are available for researchers to interpret their findings. For example, GO biological process, molecular function, and cellular component pathways of the EWAS hits can be explored by PANTHER (protein annotation through evolutionary relationship) tools (Mi et al., 2013). Other commonly used tools include Gene Set Enrichment Analysis (GSEA) (Subramanian et al., 2005), where a predefined set of genes represent a pathway collected from multiple databases such as KEGG (Kyoto Encyclopedia of Genes and Genomes) (Kanehisa et al., 2017). The commercial Ingenuity Pathway Analysis (IPA^©^ QIAGEN) can be also used to examine biological networks, functions, and associated diseases (Kramer et al., 2014). In addition to these gene centered analyses, genome region enrichment analysis has been proposed to infer the functional significance of the epigenetic marks at potential regulatory elements. For example, the LOLA tool can test a non-coding genomic region of interest for overlap with curated region set databases (Sheffield and Bock, 2016). The GREAT tool (Genomic Regions Enrichment of Annotations Tool) associates *cis-*regulatory regions identified by, e.g., ChIP-seq with biological processes by computing the enrichment scores for a given ontology term of the nearby genes (McLean et al., 2010). As a result, insights into the functional significance of the *cis-*regulatory regions across the genome are produced.

#### Chromatin States

To infer the chromatin states from epigenetics data, network-based methods such as hidden Markov model (HMM) have been developed to determine the probability of chromatin states at different genomic regions from the histone modification marks (de Pretis and Pelizzola, 2014). Notably, a widely applied method is ChromHMM which can efficiently learn the hidden chromatin states based on the distinctive combinatorial and spatial patterns of histone modification marks (Ernst and Kellis, 2017). These data-driven chromatin states are then annotated by their putative functions, such as transcription start sites, enhancers or promoters. Annotating the genome with such predicted chromatin states together with other genomic information may reveal functional elements, particularly for those regions that are in linkage disequilibrium (LD) with disease-associated SNPs. ChromHMM has been implemented in an ENCODE study to integrate 14 epigenetic marks, including histone modifications, transcription factors and chromatin accessibility for 6 human cell types, resulting in 25 chromatin states that are predictive of RNA transcription (Hoffman et al., 2013). The resulting gene regulatory elements mapped by these computational methods from ENCODE and other consortium projects have allowed individual researchers to interrogate and interpret their EWAS findings. Furthermore, computational methods that aim to predict tissue or cell-type specific functional regions have been proposed. For example, a web-based tool eFORGE (experimentally derived Functional element Overlap analysis of ReGions from EWAS) can be used to inform which trait-associated methylation hits are likely functional in a given tissue or cell type. The eFORGE method computes an enrichment score based on the overlap between the CpG sites of interest and DNase I hypersensitive sites (as marks for active chromatin) to predict the functionality of a CpG site in a given cell type, and thus help prioritize the EWAS results in terms of functional impact (Breeze et al., 2016). Another complementary method called dCMA is based on differential chromatin modification analysis to identify cell-type specific regulatory elements from ChIP-Seq data (Chen et al., 2013).

#### Gene Expression

The association between epigenetic marks and gene expression has been extensively studied to identify the functional consequences of epigenetic marks identified in an EWAS. This is commonly accomplished by linear regression models with the expression level of a gene as the dependent variable and CpG site methylation or histone modification as the independent variable. Adjusting for biological and technical confounders is also common practice in such models, which can be used to explore how epigenetic marks interact with gene expression throughout the genome. For example, a recent study in human blood cells applied a linear mixed effects model, by which DNA methylation signatures for more than 13k transcripts were defined (Kennedy et al., 2018).

While the association between CGI promoter methylation and gene expression is well-established and readily interpretable (Cedar and Bergman, 2012), the regulatory role of DNA methylation outside CGIs, in ‘shores’ and ‘shelves’ and throughout gene bodies is less extensively studied. However, methylation in these regions is potentially more relevant to diseases, as these are the regions that vary the most between tissue types and between cancerous and normal tissue (Irizarry et al., 2009). Unlike promoter methylation which is associated with gene repression, the association between intragenic methylation and gene expression is more bell-curved, with high methylation associated with moderately expressed genes and low methylation observed in genes with either high or low expression (Jjingo et al., 2012). This complex relationship between DNA methylation and gene expression poses challenges for comprehensively integrating gene expression and DNA methylation data. Public databases such as the Gene Expression Omnibus (GEO^2^) can also be employed to assist in the interpretation of EWAS findings. Inferring causal relationships between DNA methylation and gene expression can be obtained by including genetic data in the models, as discussed in the next two sections.

### Identification of Genetic Drivers of Epigenetic Marks

One of the major objectives in epigenetic studies is to identify SNPs that are associated with DNA methylation marks as meQTLs. In order to demonstrate whether trait-associated DNA methylation is independent of genetic variants influencing methylation, a regression analysis can be conducted using for example R package MatrixEQTL (Shabalin, 2012). Results of meQTL analyses include a ranked list of both short distance *cis* and more distal (>1 Mb from the DNA methylation site) *trans* effects of genetic variants on DNA methylation. Public repositories such as the mQTLdb database (Gaunt et al., 2016) and BIOS QTL browser (Bonder et al., 2017) are invaluable in epigenetic research as they enable the results from large-scale individual studies to be incorporated in subsequent meta analyses. Recently, meta-databases have been developed to systematically curate, harmonize and integrate meQTL data across different diseases. For example, Pancan-meQTL provides the result of meQTLs for 23 cancer types (Gong et al., 2018). The findings of meQTL analyses can be coupled with eQTL results in interpreting GWAS hits, as demonstrated in a recent study which identified a strong correlation between meQTLs and eQTLs that are shared by common genetic variants from peripheral blood (Pierce et al., 2018). Similar conclusions have been made in a study involving 3,841 Dutch individuals, where disease-associated variants have been found to affect both transcription factor levels and methylation of their binding sites (Bonder et al., 2017).

Integrating epigenetic marks with genotypes can also aid in interpreting the functionality of trait-associated SNPs observed in GWAS. Therefore, computational tools to predict the functions of genetic variants can be also used for annotating the functional consequences of meQTLs. Information that has been generally considered in such prediction tasks includes sequence conservation, population frequency as well as functional genomics. Approaches such as SIFT (Kumar et al., 2009) and PolyPhen2 (Adzhubei et al., 2013) align human protein sequences to homologous sequences from the other organisms to evaluate the impact of missense variants. Such sequence conservation approaches have been extended to identify conserved elements in non-coding regions by PhastCons (Siepel et al., 2005) and GERP (Davydov et al., 2010). In comparison, tools such as VAAST also utilize population frequency information from large consortiums, i.e., the 1000 Genome project for variant prioritization. Moreover, machine learning technology has long been introduced into the functional annotation of genetic variants (see Holder et al., 2017 for a recent review). For example, the PANTHER method utilizes a HMM to capture the relationship between sequence similarity and functional similarity, based on which the functional impact of a given genetic variant can be predicted (Thomas et al., 2003). As one of the most widely used methods, the CADD method employed epigenomic information such as genomic regions of DNase I hypersensitivity and histone modifications as predictive features to train the Supported Vector Machine to predict the causal variants in the genomic regions (Rentzsch et al., 2019).

### Dissecting Causality by Mendelian Randomization and Causal Networks

While many of the above-mentioned methods help illustrate the various functions of trait -associated epigenetic marks, it is often difficult to distinguish cause from consequence. In addition, the associations are often confounded by other factors. Mendelian randomization (MR) is a special form of causal network modeling, where the causality between a potential risk factor and an outcome can be established by including the genotype data (Tang et al., 2009; Latvala and Ollikainen, 2016). To be able to establish whether an association between an epigenetic mark and a disease outcome is causal, MR utilizes a series of statistical inference rules, which start by identifying an instrumental variable from the trait-associated genetic variants. This genetic instrument must fulfill the following criteria: (1) associated with the exposure, (2) independent of any potential confounders, and (3) associated with the outcome of interest only via its association with the exposure. Since the genetic variant occurs at germline that precedes the onset of disease, reverse causality is not possible. Also, as parental alleles are randomly segregated and assorted to offspring, associations between genetic variation and the outcome of interest are unlikely to be affected by confounding factors. The principles and recent developments in MR are described in detail elsewhere (Davey Smith and Ebrahim, 2003; Davey Smith and Hemani, 2014).

Mendelian randomization has been commonly used in epidemiology, and has recently been applied to infer causality in epigenetics studies as well. Depending on the applications, epigenetics marks have been considered as either the exposure or the outcome of interest in the MR model. For example, Relton and Davey Smith provided a two-step MR framework to select the instrument variables for both the risk factors and the DNA methylation marks, so that the causality cascade from the risk factors to the disease outcome can be established (Relton and Davey Smith, 2012). Such a two-step MR framework has been recently applied to study the causal roles of DNA methylation between smoking and inflammation (Jhun et al., 2017). On the other hand, a similar stepwise MR framework has been applied to distinguish causal effects from associations between blood lipid levels and DNA methylation, where the blood lipid levels were considered as the risk factor to affect DNA methylation of white blood cells (Dekkers et al., 2016). More recently, a systematic MR study involved multiple steps to investigate the meQTLs as the instrumental variables to understand the causal effect of DNA methylation for a large variety of disease traits (Richardson et al., 2018). As a validation, majority of the candidate loci were known to affect gene expression and DNA methylation, and thus supported the validity of MR as a data-driven approach to generate plausible biological hypotheses that warrant further experimental investigation. The basic version of MR involves the use of bivariate analysis, which can be extended as a causal network inference that involves the testing of multiple instrument variables in relation to different risk factors and disease outcomes. For example, the joint likelihood method (JLIM) tests whether two risk factors share the same causal genetic variants by evaluating the similarity of LD patterns between the SNPs, which is a form of co-localization methods (Chun et al., 2017). The other co-localization methods include HEIDI (heterogeneity in dependent instrument) (Zhu et al., 2016) and coloc (Giambartolomei et al., 2014) methods, while only summary-level data is used. More recently, a method called GSMR leveraged multiple SNPs as instrument variables to test for causality between risk factors and common diseases (Zhu et al., 2018).

Alternatives of causal modeling include the causal mediation analysis, which employs a series of hypothesis testing on the conditional independence among genetic variants, exposure, and disease traits (Millstein et al., 2009). The mediation analysis infers how much the indirect causal effect of an exposure on a disease outcome is mediated by a mediator, while MR focuses on the direct causal effect of the exposure on the disease outcome using a genetic variant as the proxy (Richmond et al., 2016a). A model-based causal mediation approach is available in the mediation R package (Imai et al., 2010), which has been applied in a recent study to identify nine potential epigenetic CpG sites that may mediate the effect of prenatal famine exposure to adult body mass index (BMI), serum triglycerides, and glucose levels. Notably, these CpG sites were all located at regulatory regions which are linked to the expression of growth, differentiation, and metabolism-related genes (Tobi et al., 2018).

For a model selection perspective, both causal mediation analysis and MR can be considered as special cases of causal network modeling, which compares the likelihoods for multiple competing models about causality (e.g., reverse causality model or confounding effect model) (Burgess et al., 2015). These different statistical frameworks to test for causality of epigenetic marks are useful tools, however, it is never possible to definitively prove causality based on these methods only. Instead, any negative or positive findings should be interpreted with caution and should be supported by multiple independent approaches with different assumptions, as well as the sensitivity analyses of the measurement error, and finally to match with the available biological knowledge and experimental validation (Hermani et al., 2017; Yarmolinsky et al., 2018).

## Integrative Approaches to Understand the Role of Epigenetics in Complex Traits

To date, 10s of 1000s of genetic variants have been associated with human complex traits via GWAS. Based on the findings of twin studies, these diseases and traits are, on average, 50% heritable (Polderman et al., 2015). To be able to better explain the functions of the genetic variants, the field of epigenetics has been actively researched. Next, we will describe a few representative case studies in obesity and cancer, where the integration of genetic, epigenetic, and transcriptomic data has been a key component in understanding the disease etiology and progression. The information gained from such studies can then help inform future diagnostic biomarker and treatment strategies.

### Obesity and Associated Traits

Numerous EWAS studies have shown that BMI and obesity are associated with widespread changes in DNA methylation, most often profiled using Illumina 450K or EPIC arrays (Dick et al., 2014; Ollikainen et al., 2015; Pietilainen et al., 2016; Mendelson et al., 2017; Wahl et al., 2017; Davis et al., 2018; Dhana et al., 2018). Most of the findings are tissue specific, or shared by a few tissue types (Dick et al., 2014; Wahl et al., 2017), with some hits replicated between studies, while others appear to be more study or population specific. Many of the observed DNA methylation hits are at or near genes that have previously been related to BMI or obesity traits by genetic association, while others may reflect novel genes and pathways involved in the regulation of adiposity or obesity-related diseases (Ollikainen et al., 2015; Mendelson et al., 2017; Wahl et al., 2017).

Integration of DNA methylation data with predicted chromatin states from ENCODE data has revealed that the genomic regions associated with obesity by DNA methylation are often enriched for regulatory features (Ollikainen et al., 2015; Wahl et al., 2017). Potential functional consequences of the observed methylation alterations have been tested by correlating DNA methylation with gene expression of the nearby genes, and concomitant changes in DNA methylation and gene expression have been observed in many obesity relevant genes. Integration of DNA methylation with genotype data (as meQTLs) has been used to annotate GWAS hits, and to identify novel candidate obesity-associated genes. For example meQTLs at *KLF13* (Koh et al., 2017) and *MCR4* (Tang et al., 2017) have been shown to associate with childhood obesity. In addition to identification of meQTLs, integration of genotypes and DNA methylation can be used to infer causality in the observed associations, for example by MR –based approaches. These analyses have shown that the observed associations are predominantly the consequence of high BMI or obesity – related metabolic outcomes (Dick et al., 2014; Ollikainen et al., 2015; Richmond et al., 2016b; Wahl et al., 2017). However, *NFATC2IP* and *SREBF1* methylation have been shown to have potential causal associations with BMI (Mendelson et al., 2017; Wahl et al., 2017). Finally, some studies have shown that the disturbances in DNA methylation predict future development of type 2 diabetes (Wahl et al., 2017) and coronary heart disease (Hedman et al., 2017), and that DNA methylation could be used to distinguish metabolically unhealthy from healthy obesity (Ollikainen et al., 2015; Wahl et al., 2017). To enable early detection of individuals with increased risk for metabolic complications, further studies are needed to thoroughly examine whether DNA-methylation could serve as a biomarker for metabolically unhealthy obesity.

Taken together, results from multiple epigenetic studies using data integration approaches in obesity and related traits may provide new insights into the biological pathways influenced by adiposity. Although most of the epigenetic changes are consequential to obesity or related traits, a few appear to have a causal role. Identification of causal hits is critical not only for understanding the biological mechanisms in the development of obesity and metabolic disturbances, but also for developing novel, effective prevention, and treatment strategies that target the underlying mechanisms. However, the cross-sectional nature of most of the analyzed data sets limits definitive causal determination. In addition, the marks that are caused by obesity can be considered as potential biomarkers of obesity or related metabolic disturbances. These may enable development of new strategies for prediction and prevention of adverse metabolic consequences of obesity.

### Cancer

Despite the fact that cancer has been traditionally perceived as a genetic disease, epigenetic mechanisms have been increasingly identified to contribute to many hallmarks of cancer (Flavahan et al., 2017). Epigenetic alterations are shown to be responsible for the activation of cancer oncogenes or the inactivation of tumor suppressors (Kagohara et al., 2018). Numerous recent cancer epigenetics studies have demonstrated that data integration not only enables a more detailed understanding of disease mechanisms at the molecular level, but also offers novel insights on improved approaches for disease diagnostics, treatment, and management. For example, The Cancer Genome Atlas (TCGA) project has produced DNA methylation data for over 10000 cancer samples (Hoadley et al., 2014). Here, we highlight a few representative cancer epigenetic studies where a combination of multiple data analysis methods have been applied.

One case study implemented a genome-wide chromatin accessibility profiling for chronic lymphocytic leukemia (CLL) patient samples using ChIPmentation and RNA-seq profiling (Rendeiro et al., 2016). Using a Random Forest machine learning method (Rahman et al., 2017), it was found that epigenetic profiles can accurately predict the *IGHV* mutation status. Furthermore, common and constitutively accessible regions as well as regions with higher inter-individual variability were also found. Similar studies were done using reduced representation bisulfite sequencing (RRBS) for Ewing sarcoma, a rare cancer that is known to be caused by the *EWS-FLI1* fusion gene. Despite the common genetic background, substantial DNA methylation differences between and within cancers were found (Sheffield et al., 2017). Notably, several computational tools have been developed in this study. For example, a MIRA score has been derived to transform the epigenetic state of a given genomic region into the degree of regulatory activity. Moreover, the intra-tumor heterogeneity has been measured using the PIM (proportion of sites with intermediate methylation) and PDR (proportion of discordant reads) scoring which can capture the cell-to-cell heterogeneity and the epigenetic instability within the tumor cells separately. The PIM score was then used to predict the metastatic state of a patient-derive sample using a logistic regression model.

Another study focused on triple-negative breast cancer (TNBC) by jointly contrasting the transcriptomic and epigenetic profiles of cancer stem cells (CSCs) versus non-cancer stem cells (NCSCs) (Li et al., 2018). Differentially expressed genes between CSCs and NCSCs were first identified by performing an RNA-Seq data preprocessing using tools including HTSeq (Anders et al., 2015) and samtools (Li et al., 2009), as well as differential analyses using R packages including DEGSeq (Wang et al., 2010). Subsequently, functional significance of *cis*-regulatory regions were analyzed with the GREAT (McLean et al., 2010) for the identification of significantly disrupted signaling pathways. Furthermore, patterns of differential DNA methylation and histone modifications were analyzed. By performing a WGBS analysis, differentially-methylated CpG sites in promoter regions [defined around genes’ transcription start sites (TSSs)] were identified using the methylKit R package (Akalin et al., 2012) and PeakAnalyzer (Salmon-Divon et al., 2010). In parallel, histone modifications were analyzed using ChIP-seq to determine and visualize different binding sites of antibodies specific to H3K4me2 (considered as a permissive mark for transcription) and H3K27me3 (a transcriptional silencer), using the R packages DiffBind (Ross-Innes et al., 2012) and seqMINER (Zhan and Liu, 2015). As a result, the repressive mark H3K27me3 appeared to contribute more to the tumor-promoting tendencies of CSCs, notably by affecting melanogenesis, Wnt, and GnRH pathways, all of which are known to be involved in cellular proliferation and self-renewal, conferring to the typical characteristics of chemo- or radiotherapy- resistance.

In a study conducted on epithelial ovarian cancer (EOC), the integrated analysis of genetic (GWAS), expression (proteomic) and epigenetic (DNA methylation) data permitted the identification of a novel subtype-specific susceptibility gene for the malignancy (Shen et al., 2013). As a first step, a GWAS study for ovarian cancer (consisting of 43 smaller studies and a total of more than 16,000 EOC patients) identified various *HNF1B* SNPs for the serous and the clear cell subtypes of EOC. Specifically, while rs7405776 [minor allele frequency (MAF) = 36%] was the most strongly associated SNP with serous EOC and conferred an increased risk of 13% per minor allele, rs11651755 (MAF = 45%) was strongly associated with the clear cell subtype of EOC and decreased the malignancy risk by 23% at genome-wide significance. This detection of HNF1B as a risk gene encouraged a more detailed evaluation of its promoter methylation profiles and its proteomic expression levels. An epigenetic silencing of *HNF1B* by DNA methylation was confirmed in half of the cases in the TCGA data including 576 primary serous EOC samples. To follow-up on the functional effects of the retained DNA methylation, a third cohort of 1149 EOC samples from the Ovarian Tumor Tissue Analysis (OTTA) Consortium (Bolton et al., 2012) was assessed. DNA-methylation analysis was also performed on 254 serous cases and 17 clear cell cases from those samples, using the Illumina 450K assay, with plate normalization using a linear model on the logit-transformed beta values. The correlation between the gene expression and methylation was in line with the previous hypotheses, revealing a high *HNF1B* expression and absence of promoter-methylation in most of the clear cell EOV samples, while the majority of serous samples displayed high promoter-methylation and stained negative for *HNF1B* in the IHC assay. Such an integrated analysis involving multiple omics data provides strong evidence that different genetic or epigenetic variations within the *HNF1B* gene can predispose to different histological variants of EOV, and that those variations could potentially be used as diagnostic tools for ovarian tumors.

### Epigenetics Biomarker and Drug Discovery

Upon the validation of its functional role in the disease etiology, an epigenetic mark can be further developed as a diagnostic biomarker or a drug target. By definition, a biomarker is any characteristic that can be quantified and evaluated as an indicator of normal or pathogenic biological processes, or as a measure of response to some form of treatment. Biomarkers can take a wide variety of forms, including (but not limited to) genomic modifications, RNA transcripts, proteins, and/or epigenetic alterations (Costa-Pinheiro et al., 2015). Ideally, a suitable biomarker is a highly accurate one that can be obtained in a minimally invasive or non-invasive manner, which can be utilized for screening and detection methods, diagnosis and prognostication purposes, risk assessment, and/or for the prediction of response to therapy. Accordingly, epigenetic changes are considered among the most promising classes of cancer biomarkers, owing to their stability, potential reversibility, and ease of access. There are a few epigenetic biomarkers approved in non-invasive cancer diagnosis. For example, Cologuard has become the first FDA approved test for colorectal cancer (CRC) which involves the testing of DNA methylation levels at *BMP3* and *NDRG4*, together with the mutation status of *KRAS* and hemoglobin. More recently FDA has approved a blood-based screening test for CRC called Epi procolon. The test measures the DNA methylation level of *SEPT9*, a gene that has been found to be hypermethylated in the promoter region (Issa and Noureddine, 2017).

Currently, a rich set of epigenetic biomarkers, including non-coding RNA expression levels, aberrant methylation patterns, and histone-modifying enzyme levels, are being tested in preclinical and clinical settings. For example, a urine-based epigenetic test on the DNA methylation of three genes (*TWIST1*, *ONECTU2*, and *OTX1*) in bladder cancer has achieved superior accuracy and now progressed to a larger validation study (Velazquez, 2018). Other potential epigenetic biomarkers include *SHOX2* for lung cancer and *BRCA1* for breast and ovarian cancers (Fece de la Cruz and Corcoran, 2018). To be able to leverage the existing cancer samples in the TCGA, a recent study developed a pan-cancer bisulfite sequencing assay to measure the methylation status of 9,223 GpG sites in plasma cell-free DNA in 34 major cancer types (Liu et al., 2018). The derived methylation signatures were then used for training a cancer type -specific classifier, each of which consisted of a unique set of CpG sites. The resulting classifier was used to predict the cancer type for a given sample, based solely on its methylation signature, demonstrating the feasibility of genome-wide epigenetic profiles for cancer diagnosis. In contrast, the development of epigenetics biomarkers in other disease areas is relatively in its early stage, with a few links being made for diabetes (Bacos et al., 2016) and schizophrenia (Rodrigues-Amorim et al., 2017).

In epigenetic drug discovery, histone post-translational modifications (PTMs) have been pursued as a major strategy as they constitute one of the most immediate contributors to epigenetic regulation. The PTM-affecting enzymes can be classified into three distinctive functional classes including writers, erasers and readers, which have been pursued as the targets for epigenetic drugs (Hyun et al., 2017). For example, cancer epigenetic therapy has focused on the development of targeted histone deacetylase (HDAC) inhibitors and DNA methyltransferase (DNMT) inhibitors. HDAC inhibitors activate histone acetylation, leading to higher expression of certain genes for apoptosis and cell cycle, while DNMT inhibitors re-activate tumor suppressor genes. The use of HDAC (e.g., vorinostat, belinostat, panobinostat, and romidepsin) and DNMT inhibitors (e.g., azacytidine and decitabine) has been approved for hematological malignancies. Furthermore, combinations of HDAC and DNMT inhibitors have shown synergistic interactions in a variety of cancer cell lines (Brocks et al., 2017).

In addition, overexpression and activity of histone methyltransferases (HMT) have been reported in a variety of cancers, notably acting via the silencing of essential tumor-suppressors (Bracken et al., 2003; Kim and Roberts, 2016). Consequently, HMT inhibitors such as tazemetostat and CPI-1205 have found their way to clinical development. It is unlikely that any single drug targeting epigenetic modifications is capable of curing a malignancy on its own. The combination with other such drug or with standard chemotherapeutic approaches offers the most promising prospects. For example, DNMT and HDAC inhibitors are thought to open up the chromatin conformation, thus rendering DNA more accessible to, and thereby more susceptible to damage, by chemotherapy. This observation has been validated by the successful combinations of azacitidine and low-dose cytarabine for AML (Radujkovic et al., 2014), or those of vorinostat and carboplatin or paclitaxel in non-small cell lung cancer (Owonikoko et al., 2010).

Other epigenetic modifiers that target the downstream proteins also have sparked interest. For example, the family of bromodomain containing proteins known as BETs have been involved in chromatin remodeling and transcriptional activity in a variety of diseases including inflammation, viral infection and cancer (Ferri et al., 2016). Furthermore, BET inhibition has been shown to decrease *MYC* expression and to restore normal cellular functions in a variety of cancers including hematological malignancies and solid tumors (Wang and Filippakopoulos, 2015). The first potent and selective BET inhibitor is the thieno-tiazolo-1,4-diazepine, known as the positive enantiomer (+) of JQ1. Other BET inhibitors include I-BET762 which is currently being investigated in several ongoing clinical trials for different cancers (Andrieu et al., 2016).

### Pharmacoepigenetics

Due to the lack of full annotations on the drug-induced epigenetic changes, the exact mode of action of the epigenetic drugs in different cancer cells remains largely unknown, which partly explains the individual variation in the clinical response (Treppendahl et al., 2014). On the other hand, it has been shown that many common drugs also induce epigenetic changes via the direct interaction with the PTM-affecting enzymes, or the downstream drug signaling pathways (Lotsch et al., 2013). These epigenetic changes may contribute to both the therapeutic and the adverse effects of the compounds, which are also mediated by the patient’s individual genetic background, e.g., of drug-metabolizing enzymes and transporters. Only recently the concept of pharmacoepigenetics has started to emerge, aiming at the study of epigenetic mechanisms to explain the interindividual variability in drug responses (Majchrzak-Celińska and Baer-Dubowska, 2017; Lauschke et al., 2018). The epigenetic regulators of drug responses have been often linked to ADME (drug absorption, distribution, metabolism, and excretion) genes. For example, many genes in the Cytochrome P450 family are reported to be directly or indirectly regulated by miRNAs (Kim et al., 2014). Hypomethylation of the *ABCB1* promoter region has been shown to increase the gene’s expression in cancer cells, leading to acquired drug resistance (Reed et al., 2010). Research in this field may eventually lead to the development of ADME-related biomarkers for the stratification of patients into different treatment groups. In addition, epigenetic biomarkers that are not linked to ADME genes were also reported, while the exact mechanisms remain largely undetermined. In breast cancer for example, the quantification of *PSAT1* DNA methylation is used to predict tamoxifen response (Martens et al., 2005; De Marchi et al., 2017), whereas that of *BRCA1/2* (similarly to somatic mutations in those genes) is indicative of response to PARP inhibitors (Martens et al., 2005). Similarly, hypermethylation of *MGMT* and *MLH1* correlates with increased response to 5-FU treatment and improved survival in CRC (Nagasaka et al., 2003; Jensen et al., 2013). Notably, a recent clinical study has discovered a DNA methylation signature to predict the response of Anti-Programmed Death-1 (PD-1) treatment for advanced non-small-cell lung cancer (Duruisseaux et al., 2018). Another clinical study called Genetic and Environmental Determinants of Triglycerides (GOLDN) measured the genetic and epigenetic profiles for metabolic syndrome using a family-based design (Aslibekyan et al., 2018). In this study, the epigenetic profiling was made before and after the treatment of fenofibrate, allowing the characterization of genotype and DNA methylation to understand the variability in the drug treatment response. Despite that potential biomarkers have been found in these recent advances, a systematic strategy to predict and understand the epigenome-wide interactions mediating the drug responses is still lacking. We anticipate that data integration methods as summarized in previous sections that are capable of annotating the epigenome from a pharmacological and pharmacokinetic perspective shall provide a valuable source of information to inform personalized treatment decisions.

## Conclusion

Understanding epigenomic regulation is critical for dissecting gene–environment interactions in both normal development and disease. The fact that epigenetic profiles are plastic and reversible holds great promise for developing epigenetic biomarkers and drug targets. Furthermore, epigenetics captures the spatial and temporal variation on top of each individual’s unique genome, and thus better informs the decision-making in personalized medicine. Recent developments have made chromatin accessibility profiling more cost-effective by allowing only a small number of cells as input, demonstrating the clinical potential of disease monitoring (Buenrostro et al., 2015). On the other hand, biobanks have made large scale clinical samples accessible and often provide functionality to share the accumulating raw data and molecular profiles similar to the concept of European Genome-Phenome Archive (EGA) (Lappalainen et al., 2015). Although individual epigenetic marks are often studied in isolation, the understanding of how the putative gene regulatory mechanisms occur will not be achieved without efficient tools to design, analyze, integrate, and interpret the versatile epigenetic features. To facilitate the systematic characterization of cells in a specific context, the other omics data such as transcriptomics and metabolomics may also provide complementary information to explain the interplay of the gene–environment interaction. Further developing the data integration tools shall more efficiently prioritize robust epigenetic modifications that are susceptible to environmental exposures and causal to specific diseases, so that specifically targeted compounds can be developed. Furthermore, despite the advances in these computational methods, one needs to ultimately resort to experimental approaches to confirm the hypothesis. The recent development of CRISPR-Cas9 and other genome editing tools may provide an efficient way to induce epigenetic alterations without the change of DNA sequences, so that novel drug targets and disease biomarkers may be identified more efficiently (Liao et al., 2017).

## Author Contributions

EC, CH, MO, and JT conceived the study. All authors participated the writing of the manuscript.

## Conflict of Interest Statement

The authors declare that the research was conducted in the absence of any commercial or financial relationships that could be construed as a potential conflict of interest.

## Footnotes

1 https://www.nature.com/collections/sqtqxdnvdz

2 https://www.ncbi.nlm.nih.gov/geo/
